# 

**Published:** 1919-04-26

**Authors:** 


### Exhibition of Work at St. Mary's.

An exhibition of work was held in the Board-room of St. Mary's Hospital by members of the Ladies' Association of Paddington. There were 500 garments, which are always most acceptable, articles of linen, and from the Anglo-South American Association garments and dressings. Many visitors were present, as well as many contributors, who year by year work for this annual contribution. The wards were thrown open for inspection. Tea was served.

### GIFTS AND BEQUESTS.

Sir Ernest Cassel has sent a donation of £1,000 to the Church Army.

Mr. W. Arthur Sturdy, of Hayward's Heath, left £3,000 to King Edward's Hospital Fund.

Mr. Henry Alfred Jones, of Eastbourne, left £500 to the London Hospital and £250 each to seventeen hospitals in London and the Provinces.

Sir Mark Oldroyd has given £10,000 to Dewsbury Infirmary, and £2,500 to Dewsbury Day Nursery, in memory of the late Lady Oldroyd.

Mr. John Thomas Smith, of Atherton, left £500 to the Manchester Royal Infirmary.

Mr. H. Bas[illegible]am Dickinson, of Liverpool, left an annuity of £25 to the Essex County Hospital.

Mr. Herbert W. Hind, of Bidston, Cheshire, bequeathed £500 each to the Birkenhead Borough Hospital and the Wirral Children's Hospital.

Mr. John Lind, of Liverpool, has given £1,000 to endow a bed in the Hahnemann Hospital, Liverpool, in memory of his son, Second-Lieutenant J. V. Lind.

### Guy's Hospital Employees.

Now that the war is over, the employees of Guy's Hospital have turned their attention towards the reconstruction of the social side of hospital life. In 1915 a social club was started by the officers and servants of the hospital, but only had a brief career, as so many of the male staff enlisted in the Forces. At a meeting held on April 8 it was impressed upon all those present that the object of the Committee was to run the club on purely democratic lines, so that every hospital officer and servant could meet on an equal social basis. Mr. Croucher (Medical School) stated "that after all we were all servants of Guy's Hospital and proud of the institution we serve." It was also pointed out that it provided the missing link connecting the various departments of the hospital and medical school. In proposing "that lay members of the lady administrative staff be admitted," one member said it was necessary to move with the spirit of the times, and as there was now a much larger staff of ladies, he thought that they should be invited to co-operate. It was decided to carry on very quietly through the summer months with a Rambling Club and a social or two, then call a general meeting and extend a cordial invitation to all officers and servants to attend.

